# Future continental summer warming constrained by the present-day seasonal cycle of surface hydrology

**DOI:** 10.1038/s41598-020-61721-9

**Published:** 2020-03-13

**Authors:** F. M. Selten, R. Bintanja, R. Vautard, B. J. J. M. van den Hurk

**Affiliations:** 1 Royal Netherlands Meteorological Institute (KNMI), De Bilt, The Netherlands; 2 Energy and Sustainability Research Institute Groningen (ESRIG), University of Groningen, Groningen, The Netherlands; 3 Laboratoire des Sciences du Climat et de l’Environnement (CEA/CNRS/UVSQ), Institut Pierre-Simon Laplace, Gif sur Yvette, France; 4 Deltaris, Delft, The Netherlands; 5 Free University of Amsterdam, Amsterdam, The Netherlands

**Keywords:** Atmospheric dynamics, Climate and Earth system modelling

## Abstract

Present-day land temperatures simulated by state-of-the-art global climate models exhibit considerable uncertainty. Generally it is assumed that these temperature biases do not affect the projected warming in response to rising greenhouse gas concentrations (i.e. drop out by subtracting projected and present-day temperatures), but for specific regions and seasons this assumption is invalid. Here we show that, on the contrary, for large continental regions, such as Europe, state-of-the art global climate models with a warm summer bias project a relatively strong warming. This is because continental summer temperatures depend chiefly on soil drying in response to spring and summer solar radiation increase: models that dry fastest (due to the interaction of clouds, convection and soil hydrology) exhibit the strongest reductions in evaporation and consequently a more pronounced end-of-summer warming. These physical mechanisms acting on a seasonal timescale also govern the long-term climate response to greenhouse forcing over continental regions in summer. Combining these findings, we use the current model biases to reduce the uncertainty range in the projected warming over Europe from 3.6–8.6 °C to 4.6–7.3 °C (a reduction of about 50%). Given the huge potential impacts of the warmest projections on health, agriculture and water management, constraining the range of future summer climate change is imperative for relevant mitigation and adaptation strategies.

## Introduction

Heat waves are projected to occur more often in a warming climate^1^. For example, the devastatingly hot European summer of 2003 is expected to become a normal summer in 2050 in low-mitigation scenarios^2^. Clearly, society will have to adapt to these profound changes. Accurate projections of the anticipated warming are a prerequisite to take timely and appropriate measures with respect to public health (e.g. spread of diseases), water management and agriculture, but they rely on a thorough understanding of the underlying physical processes. A number of mechanisms have been proposed that contribute to, or amplify, continental summer warming and heat waves: (1) depletion of soil moisture which limits surface evapotranspiration, with the loss of evaporative cooling amplifying near-surface warming^3,4^ with potential non-local effects^5^, (2) a diminished evaporative water vapour flux reduces cloud amount, causing the incident solar radiation at the surface to increase, which further warms the near-surface atmospheric layers^6^, (3) strong local warming may cause the formation of a so-called local heat-low (for instance over Southern Europe), which induces stronger easterly winds and advection of warm and dry continental air masses into Central Europe, further enhancing the warming^7^. Clearly, several climatic processes and feedbacks potentially affect summer continental warming, of which the magnitudes are as of yet unknown.

Given this variety of processes and feedbacks involved in continental summer warming and the fact that models generally differ in the representation of these processes as well as in their mutual interactions, model-based projections such as those from the CMIP5 (Climate Model Inter-comparison Project, phase 5) initiative exhibit a considerable summer-warming spread^8^, indicating that the associated uncertainties are substantial. Moreover, the simulation of present-day temperatures shows a large inter-model spread, implying that climate models, despite having been improved drastically over the past 30 years, still exhibit considerable biases with respect to the current climate^9^. Even though it is assumed that, overall, these biases do not affect projected warming^10^, temperature biases in certain regions/season increase with temperature, which allowed to statistically link bias corrections to the magnitude of projected summer warming^11^. Direct observations of surface heat flux have been used to constrain uncertainties in projected warming, without specifically addressing the physical mechanisms involved^12^. On the basis of statistical relations of summer only data it has recently been suggested that there is a relation between the warming and the summer bias^13^. Future continental summer warming is therefore still not well constrained in the sense that uncertainties remain considerable and that the associated mechanisms and their individual contributions are still largely unknown.

**Figure 1:**
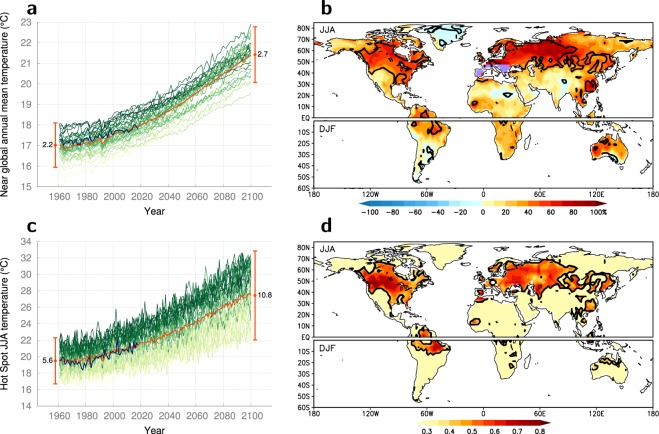
Surface air temperature simulations. (**a**) Time-series of near-global (60°S–70°N) annual mean temperatures for 31 CMIP5 climate models under the RCP8.5 scenario (various green colors correspond to each models’ 1961–1990 mean temperature, the darker the warmer), for the ensemble mean (orange), and for the observations (dark blue). The length of the vertical lines indicates the spread among the models during the first and the final 30 years respectively and corresponds to four times the standard deviation around the ensemble mean temperature (centred on the ensemble-averaged temperatures). (**b**) Fractional increase in the spread of the simulated land temperatures among the models between present (1981–2010) and future (2071–2100) for both summer hemispheres. The black contours indicate regions where the increase in spread is larger than the expected increase in spread under the hypothesis of no relation between the simulated warming and the simulated present-day temperatures (see Methods for details). (**c**) Time-series as in (**a**) but for temperatures averaged over the hot-spot region, indicated by the purple area in (**b**) and defined as the region in Europe where the ensemble mean summer warming is in excess of 6 K. (**d**) Inter-model correlation between present-day temperature and warming. Within the black contours the correlation coefficients are significant at the 95% confidence level.

Here we focus on the crucial physical processes in the present-day seasonal cycle as simulated by 31 state-of-the art CMIP5 global climate models to elucidate the origin of the relation between present-day summer bias and projected warming. Using this relationship we constrain the projected summer warming. We use standardised simulations for the period 1961–2100 based on the Representative Concentration Pathways scenario 8.5 (RCP8.5). Simulated present-day global annual mean temperatures exhibit an inter-model range of 2.2 °C, which is large compared to the mean projected warming of about 4 °C (Fig. 1a). This range increases by about 25% into the future. However, there are considerable regional differences in the increase in inter-model temperature range (Fig. 1b), with maximum values over mid-latitude continental summer regions and peak increases of up to 80% over Southern Europe. These are also the regions where the projected summer warming correlates with the present-day temperature bias with warmer models projecting stronger warming (Fig. 1d, Supplementary Table 1). Southern Europe is the region with most vigorous projected summer warming of over 6 °C (Fig. 1b,d, purple area). Because of the potentially large societal impact of summer warming in Europe, we will henceforth focus on the reasons behind the excessive summer warming in this hot-spot region. The inter-model temperature range in the hot-spot region increases from 5.6 °C in the present-day to 10.8 °C in the future (Fig. 1c), demonstrating that the uncertainty in summer temperature projections on this regional scale is much larger than its global counterpart (Fig. 1a). Quantifying and understanding the governing physical mechanisms underlying the range in projected regional warming is a prerequisite for deciding which projections are more reliable.

**Figure 2:**
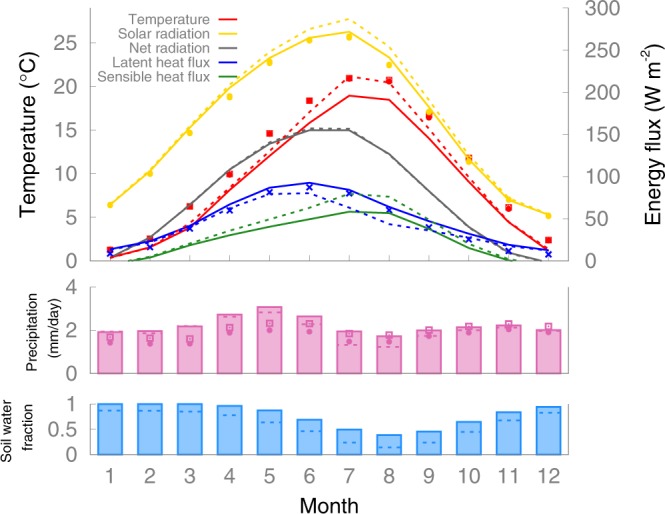
Mean seasonal cycle during 1981–2010 averaged over the hot-spot region as simulated by the EC-Earth climate model. Solid lines indicate surface air temperature (red), downward solar heat flux at surface (yellow), net absorbed radiation at surface (black), upward latent heat flux (blue) and upward sensible heat flux (green). Pink bars indicate total precipitation, blue bars the amount of soil water available for evaporation as a fraction of the maximum amount of soil water that can potentially evaporate from the upper two layers of the land model with a combined depth of 28 cm (equal to the field capacity minus the wilting point). Dashed lines represent corresponding values for a sensitivity simulation with a more efficient surface runoff (see Methods). Symbols indicate observational estimates averaged over the same area and period (dots corresponds to the E-OBS v20.0e dataset, squares to CRU TS v4.03 and crosses to GLEAM v3.3a).

End-of-summer temperatures over continental Europe depend heavily on the availability of soil moisture for evapotranspiration. In response to the seasonal march of solar radiation (Fig. 2), evapotranspiration starts to increase in springtime, thereby cooling and drying the land surface. Soil moisture is depleted from May into summer and it is limiting evapotranspiration in July, even though surface solar radiation still increases. Evapotranspiration capping increases the sensible heat flux and surface air temperatures continue to rise. In addition, reduced evapotranspiration leads to fewer clouds and thus more solar radiation reaching the surface, thereby reinforcing the end-of-summer warming. The crucial physical processes that play a role in determining the end-of-summer temperatures include the transport of water in the soils, root uptake of water and evapotranspiration in vegetation, atmospheric convection, the formation of clouds, the effect of clouds on radiation, precipitation and the large-scale transports of air masses. Since these (often fine-scale) processes and their mutual interactions are hard to model accurately (they are often parameterised in course-resolution climate models), it is not surprising that models suffer from large biases in end-of-summer temperatures and that model-based projections of summer temperatures in continental Europe are subject to large uncertainties.

**Figure 3:**
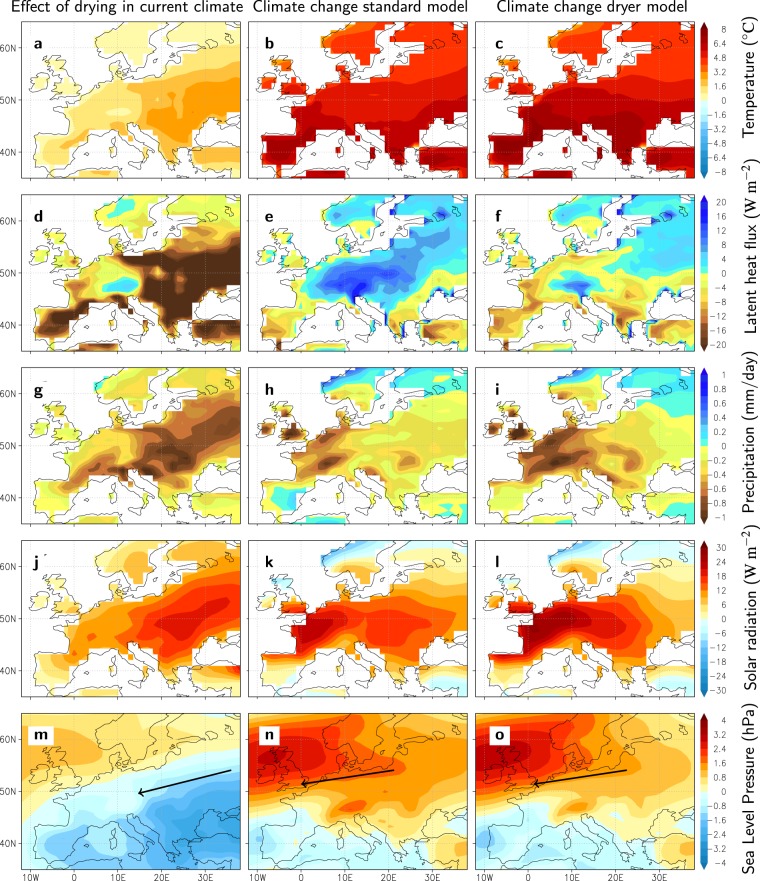
Simulated effects of imposed enhanced soil drying on European summer climate (change). The first column shows the differences in the current climate (1981–2010) between the simulation with a more efficient surface runoff (see Methods) and the control simulation using EC-Earth. The second and third columns depict the climate change between 2071–2100 and 1981–2010 for the control simulation and for the more efficient surface runoff simulation, respectively. Panels (**a–c**) show surface air temperature (°C), (**d**–**f**) surface evaporation expressed as the surface latent heat flux (W m^−2^), (**g**–**i**) total precipitation (mm day^−1^), (**j**–**l**) net solar radiation at surface (W m^−2^) and (**m**–**o**) mean sea-level pressure (hPa) with arrows indicating the implied wind change.

We hypothesise that the end-of-summer warming in response to increased greenhouse forcing is driven by the same processes and their mutual interactions as those driving the seasonal warming in response to the seasonal march of solar radiation, thereby explaining the relation between bias and warming. We use one of the CMIP5 models (EC-Earth^14^) in climate warming sensitivity experiments (imposing a fraction of the precipitation to directly drain into rivers, forcing dryer soils, see Methods) to test whether a simple adjustment in the surface run-off parameterization leads to a process-based response in the present-day seasonal cycle that is similar to that in projected changes. Over Europe, the imposed drying leads to warmer summers in the current climate as a result of reduced evaporative cooling (Fig. 3a,d). Positive feedbacks further enhance the response as reduced precipitation and cloud cover cause enhanced solar radiation reaching the surface (Fig. 3g,j), while easterly advection of warm and dry air masses occurs along the northern flank of a surface heat low that develops over the area of strongest warming (Fig. 3m). In the non-perturbed control simulation the evaporative cooling continues to increase into the future, indicating that soil moisture availability is not a limiting factor (Fig. 3e). In contrast, in the simulation with enhanced surface runoff and dryer soils, the future evaporative cooling is limited due to the imposed depletion of soil moisture (Fig. 3f), with consequent larger sensible heat fluxes leading to amplified warming (Fig. 3b,c). In addition, the warming in the dryer simulation is enhanced by the same positive feedbacks operating on the seasonal cycle, namely a stronger reduction in precipitation (Fig. 3h,i), an increase in solar radiation (Fig. 3k,l) and an intensified easterly advection of warm and dry air masses (Fig. 3n,o).

**Figure 4:**
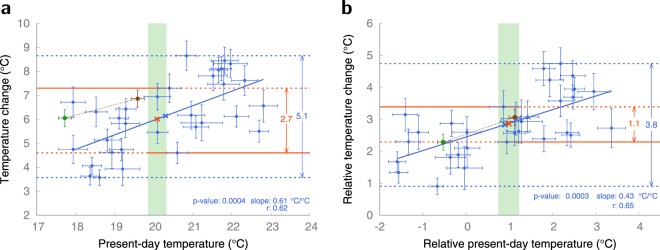
Inter-model relation between simulated present-day summer temperatures and temperature changes in the hot-spot region. (**a**) Present-day hot-spot summer temperature versus the temperature change (2071–2100 minus 1981–2010) for each of the 31 CMIP5 models (blue dots), the ensemble mean (blue cross), the EC-Earth control simulation (green dot), the EC-Earth simulation with more efficient surface runoff (brown dot) and the warming implied by the linear regression (solid blue line) given the observed 1981–2010 hot-spot temperature (orange cross). The uncertainty in the observed current temperature is indicated by green shading. The unconstrained uncertainty range in the simulated warming in the full ensemble is defined here by the models with the strongest and weakest warming (stippled blue lines) and is indicated by the vertical blue arrow. The upper bound of the constrained uncertainty range is defined by the strongest warming among models with correct or too cold temperatures, the lower bound by the weakest warming among models with correct or too warm temperatures (orange lines and arrow). (**b**) as (**a**) but for temperatures relative to the near-global mean temperature (60°S–70°N).

These sensitivity simulations show that the physical processes leading to stronger drying and warming in the present-day seasonal cycle also induce more intense future drying and amplified summer warming^15^. By imposing increased runoff and soil drying in EC-Earth, the current temperature and projected future warming in the hot-spot region both increase according to the multi-model regression line (Fig. 4a), suggesting that the inter-model relation between current state and future warming is indeed governed by soil drying and reduced evaporation. This can be used to physically explain and constrain projected future warming. The increase in uncertainty in the hot-spot region is clearly, and significantly, related to the present-day bias (Fig. 4a). Models with the warmest current biases exhibit the strongest summer warming (a bias of +1 °C results in an additional warming of 0.6 °C). Observed present-day hot-spot temperatures are in the 19.8–20.3 °C range, which corresponds to a projected warming of 4.6–7.3 °C. Hence, using this relation we have effectively reduced the projected summer temperature range by about 50%. The bias-warming relation is even more pronounced if the analysis is expressed in terms of temperatures relative to the near-global mean temperature (Fig. 4b). Using relative temperatures, inter-model differences are even more firmly tied to local physical processes contributing to the warming, since the effects of inter-model differences in global climate sensitivity are then filtered out.

Climate model projections of future summer conditions are the basis for adaptation and mitigation strategies concerning health^16^ (heat-wave-related mortality, spread of diseases), agriculture^17^, water management^18^ (e.g. shipping, drinking water, power plants cooling), wild fires^19^ and potentially vulnerable ecological systems^20,21^. Especially during summer people’s everyday experience of climate change effects are expected to become unpleasant in the near future^22^. Unfortunately, climate models exhibit biases that strongly contribute to considerable uncertainties in the projected summer changes. Here we employ a physically justified statistical relationship between the present-day seasonal cycle and the projected future changes to reduce the uncertainties of European summer warming projections by ~50%. During spring and summer, increased solar insolation evaporates soil moisture (the magnitude of which depends on the amount of clouds, amount and type of vegetation, atmospheric circulation) until depletion of soil moisture limits the surface evaporative cooling, after which the sensible heat flux amplifies the end-of-summer warming. Even though Europe was highlighted in this study, similar mechanisms clearly govern the response in other mid-latitude continental regions that exhibit strong summer warming (Fig. 1) for which the projected warming range can therefore be significantly reduced (see Methods). Present-day summer land temperatures thus serve as an “emergent constraint”^23^ on projected future warming. Evidently, models that accurately represent the seasonal soil drying and the associated end-of-summer temperature will simulate the most reliable climate projections. The change in the run-off parameterization in EC-Earth improved evaporation and precipitation in most months of the year (Fig. 2). As a result of the run-off modification, the cold temperature bias in July and August is removed, but provided we can trust the observed estimates of evaporation and global radiation, probably not entirely for the right reasons (global radiation increased too much, perhaps too few and/or not bright enough clouds, probably in response to too strongly reduced evaporation, even though net radiation did not change). EC-Earth simulates too much precipitation in springtime related to a too zonal circulation, and this precipitation bias is only partially remedied by the run-off modification. Springtime precipitation is also improved in a higher resolution version of EC-Earth^24^, in which the authors find that the reduced springtime rainfall leads to dryer summer soils, a reduced summer temperature bias and a stronger projected warming, in accordance with the findings of our study. The existence of strong feedbacks during the seasonal cycle between soil moisture, evaporation, solar radiation (through cloud changes), precipitation and circulation is unequivocally demonstrated in this study. These feedbacks largely depend on the surface run-off parameterization and determine end-of-summer temperatures as well as the projected warming. In conclusion, model developments intended to produce more credible summer warming and precipitation projections should demonstrate a more realistic representation of the present-day seasonal cycle of water and energy fluxes in regions where a temperature bias versus projected warming relationship exists.

## Methods

The model analyses are based on the Coupled Model Inter-comparison Project, phase 5 (CMIP5) state-of-the-art global climate models, which were applied in a series of standardised forcing scenario’s for the period 2006–2100, following simulations with historical forcings from 1860–2005. Here we use the RCP8.5 scenario for which the combined greenhouse, aerosol and other radiative forcings in the year 2100 equals 8.5 W m^−2^. We use 31 models for which data coverage was complete and without obvious errors (other than that no selection of models was made); one ensemble member per model (the first) was used. Present-day refers to the period 1981–2010, and the future to 2071–2100. Globally observed temperatures were taken from the HadCRUT4 dataset that includes estimates of the observational errors; evaluation of the seasonal cycle in Europe is based on E-OBS v20.0e for temperature, precipitation and global radiation, CRU TS v4.03 for temperature and precipitation and GLEAM v3.3a for land evaporation.

The sensitivity experiments were carried out with EC-Earth version 2.3, which is one of the CMIP5 models. The control experiment consists of a simulation (4 members) from 1950–2100 under the RCP8.5 scenario, each with prescribed sea-surface temperatures and sea-ice distributions taken from various coupled atmosphere-ocean simulations under the RCP8.5 scenario. The sensitivity experiment (also 4 members) only differs in the formulation of the surface runoff: a minimum fraction of 20% of the local rainfall above land is directly drained into the rivers instead of entering the soil. The effect of the surface runoff modification is analysed as the difference between the two ensemble means.

For the statistical test of the null hypothesis that model bias has no relation to projected warming we use 31 CMIP5 models. Given 31 mean summer temperatures for the periods 1981–2010 and 2071–2100, we generated surrogate 31-member ensembles of future temperatures by picking randomly a present-day temperature of model X and adding the warming of model Y. These ensembles of future temperatures are then consistent with the zero hypothesis that the present-day temperature is unrelated to the warming. We calculated the standard deviation of the future temperatures as an indicator of the spread and determined the probability density function of the spread based on 50,000 surrogate ensembles. When the true spread of future temperatures in the CMIP5 ensemble is in the tail of this distribution, we can reject the zero hypothesis and conclude that there is in fact a relation between bias and warming.

Online Content Methods, along with any additional Supplementary display items, are available in the online version of the paper; references unique to these sections appear only in the online paper.

## Supplementary information

Supplementary Information.
