# Supplementary material for: Future continental summer warming constrained by the present-day seasonal cycle of surface hydrology
Source: Sci Rep. 2020 Mar 13;10:4721. doi: 10.1038/s41598-020-61721-9 (PMC7069940; doi:10.1038/s41598-020-61721-9)
Supplement: Supplementary file 1 — Supplementary Information. [file 41598_2020_61721_MOESM1_ESM.docx]

Supplementary Information

**Future continental summer warming constrained by the present-day seasonal cycle of surface hydrology**

F. M. Selten, R. Bintanja, R. Vautard & B. J. J. M. van den Hurk

**Statistical test of the null hypothesis that model bias has no relation to projected warming**

Supplementary Figure 1 illustrates the statistical test as outlined in Methods for the annual near-global mean temperature and the summer hot-spot temperature. For the near-global mean temperature the spread of future temperatures is consistent with the null hypothesis. In some regions however, for example in the summer hot-spot region, warmer models project stronger warming, leading to a spread that is larger than expected under the null hypothesis.

**Mean seasonal cycle for the period 2071-2100 averaged over the hot-spot region as simulated by the EC-Earth global climate model.**

The effect of the run-off modification on the seasonal drying and subsequent capping of the evaporative cooling in the hot-spot region is stronger in the future than in the present-day climate, as illustrated by Supplementary Figure 2. The run-off modification reduces the evaporative cooling by 18 W m^-2^ in JJA the present-day climate, but by 24 W m^-2^ in the future climate. This mechanism thus can explain the multi-model relationship between present-day summer temperatures and projected summer warming in the hot-spot region. Note that also in the future, the net radiation absorbed by the surface is hardly affected by the run-off modification, as the increase in absorbed solar radiation is compensated by the net increase in cooling by upward thermal radiation.

**Constraining the projected warming for summer continental regions other than the hot-spot region**

This study has identified regions where models that are too warm in the present-day summer are likely to over-estimate the warming in response to the enhanced greenhouse effect. This knowledge is used in Figure 4 to constrain the warming in the hot-spot region. The unconstrained range is given by the minimum and maximum warming in the ensemble, the constrained range by the minimum warming among models with correct or warmer present-day temperature and the maximum warming among models with correct or colder present-day temperature. We also applied the same approach to other regions where according to Figure 1bd warmer models project stronger warming. These regions are located in North-America, Russia, China and Australia and are defined as land points where the fractional increase in summer temperature spread exceeds 50% in North-America and Australia, 75% in Russia and 65% in China. Supplementary Table 1 summarizes the results. In North-America and Russia the reduction in the spread of the projected warming is similar to that obtained for the hot-spot region in Europe. In China and Australia the reduction is somewhat lower because in these regions the relation between current temperature bias and projected warming is less pronounced. The consistency of the results across these regions is an important result.


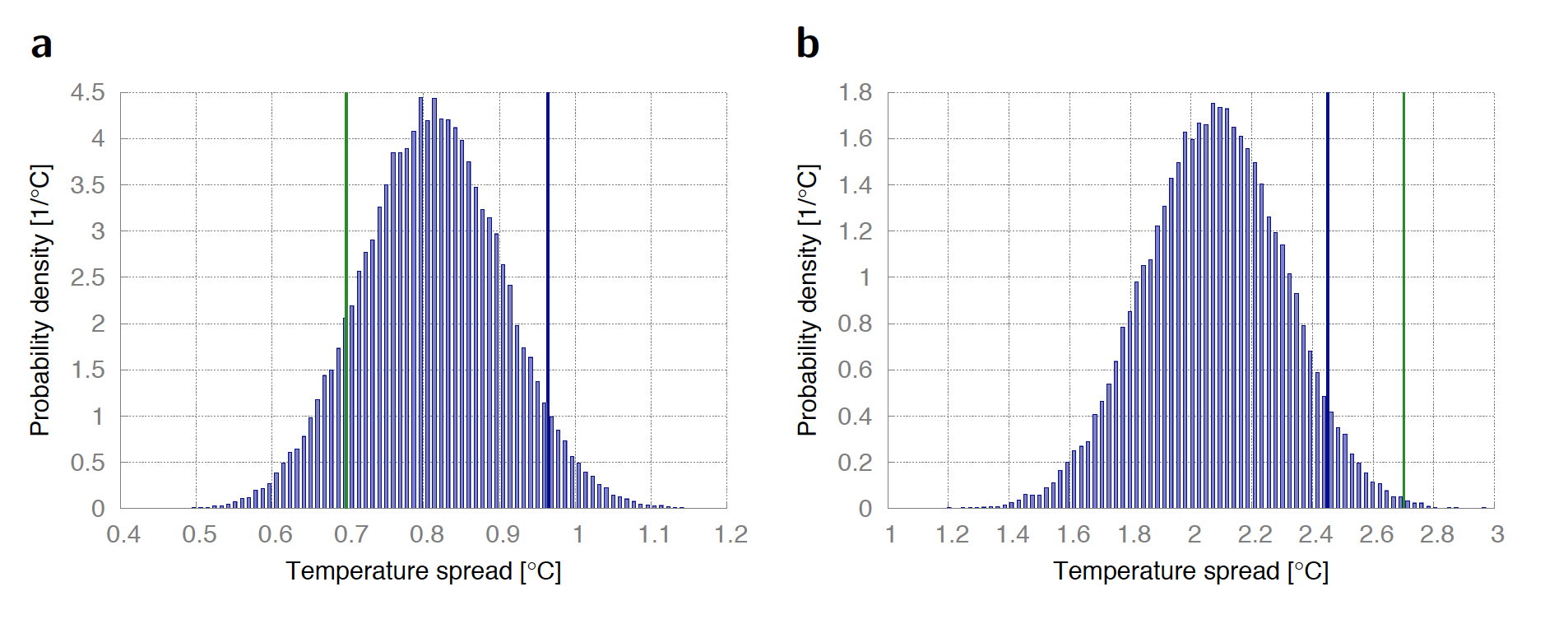


**Supplementary Figure 1 | Probability density function of the spread in future temperatures consistent with the zero hypothesis that simulated present-day temperatures exhibit no relation with the projected warming.** (a) Probability density function (PDF) of the spread in 31-member ensembles of surrogate future annual near-global mean temperatures (60° S – 70° N) constructed by randomly choosing the present-day temperature of CMIP5 model X and adding the projected warming of model Y. Here the standard deviation is used as an indicator of the spread. The PDF is estimated from 50,000 surrogate ensembles. The actual spread in the CMIP5 future temperatures is indicated by the vertical green line, the 95% percentile by the blue line. (b) as (a) but for hot-spot temperatures.

**Supplementary Figure 2 | |** **Mean seasonal cycle during 2071-2100 averaged over the hot-spot region as simulated by the EC-Earth climate model.** Solid lines indicate surface air temperature (red), downward solar radiation at surface (yellow), net absorbed radiation at surface (black), upward latent heat flux (blue) and upward sensible heat flux (green). Pink bars indicate total precipitation, blue bars the amount of soil water available for evaporation as a fraction of the maximum amount of soil water that can potentially evaporate from the upper two layers of the land model with a combined depth of 28 cm (equal to the field capacity minus the wilting point). Dashed lines represent corresponding values for a sensitivity simulation with a more efficient surface runoff.


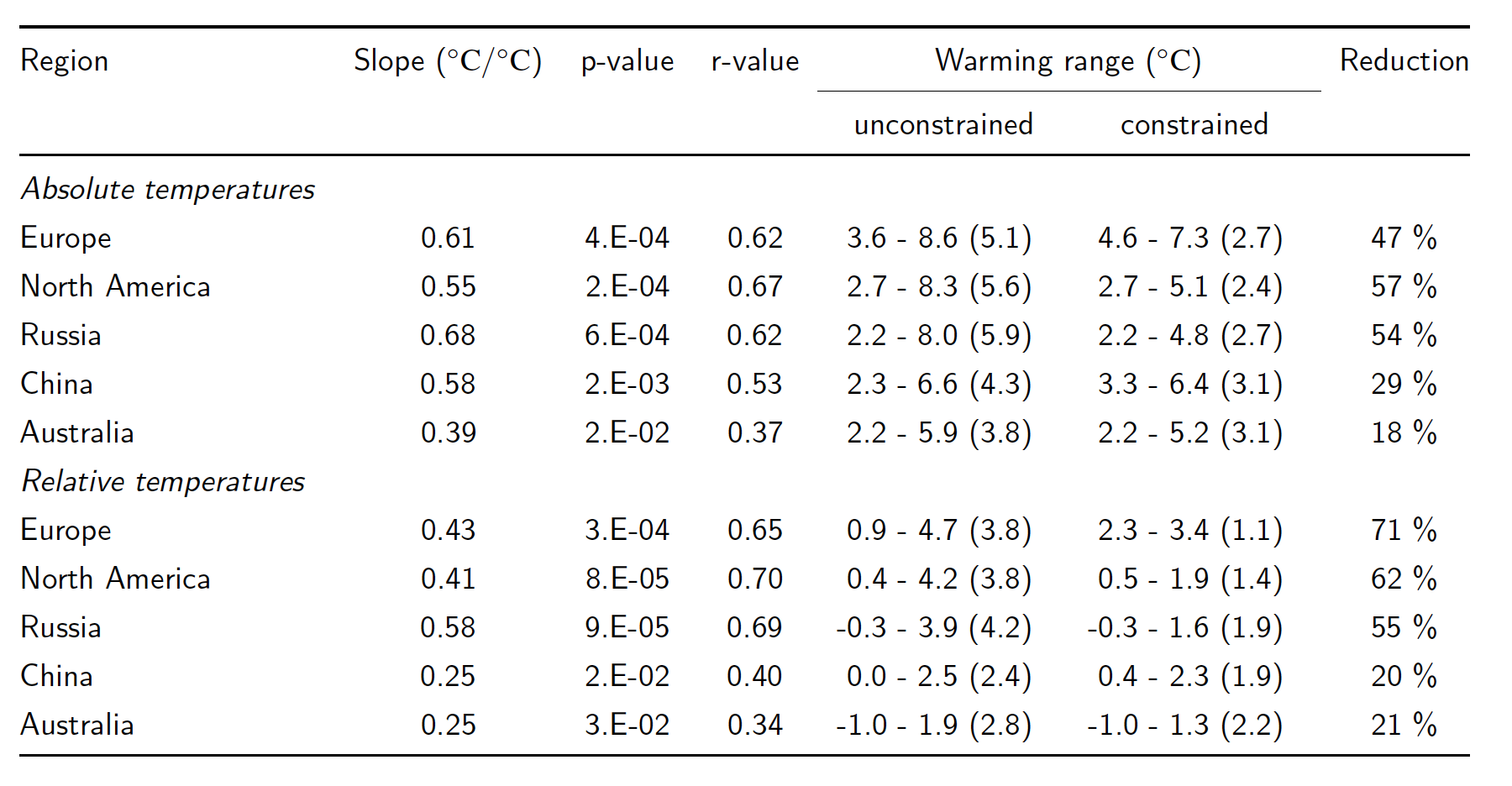


**Supplementary Table 1 | Unconstrained and constrained ranges in projected warming for the CMIP5 model ensemble under the RCP8.5 scenario in selected continental summer regions.** In all selected regions, models with warmer present-day (1981-2010) temperatures project stronger warming in the future (2071-2100). The relationship is characterised by the slope of the linear regression line with the associated p and r values. The unconstrained range is given by the minimum and maximum warming in the ensemble, the constrained range by the minimum warming among models with correct or warmer present-day temperature and the maximum warming among models with correct or colder present-day temperature. The final column depicts the relative reduction resulting from the constraining procedure. Results are shown for absolute and relative (with respect to the near-global mean [60° S – 70° N] ) temperatures.
